# Physicochemical, microbiological, and sensory properties of healthy juices containing aloe vera gel and probiotics and their antidiabetic effects on albino rats

**DOI:** 10.3389/fnut.2024.1328548

**Published:** 2024-07-16

**Authors:** Sara Naiim Moselhy, Ahmed Aladdin Al-Nashwi, Enrique Raya-Álvarez, Fouad Omar Abu Zaid, Hanan Said Tawfik Shalaby, Manal F. El-Khadragy, Magdy Ramadan Shahein, Amin A. Hafiz, Abeer A. Aljehani, Ahmad Agil, Ehab Kotb Elmahallawy

**Affiliations:** ^1^Food Science Department, Faculty of Agriculture, Zagazig University, Zagazig, Egypt; ^2^Rheumatology Department, Hospital Universitario San Cecilio, Granada, Spain; ^3^Agri- Industrialization Unit, Plant Production Department, Desert Research Center, Cairo, Egypt; ^4^Department of Biology, College of Science, Princess Nourah Bint Abdulrahman University, Riyadh, Saudi Arabia; ^5^Department of Food Science and Technology, Faculty of Agriculture, Tanta University, Tanta, Egypt; ^6^Department of Clinical Nutrition, Faculty of Applied Medical Sciences, Umm Al-Qura University, Makkah, Saudi Arabia; ^7^Department of Food and Nutrition, Faculty of Human Sciences and Design, King Abdulaziz University, Jeddah, Saudi Arabia; ^8^Department of Pharmacology, Biohealth Institute Granada (IBs Granada) and Neuroscience Institute, School of Medicine, University of Granada, Granada, Spain; ^9^Departamento de Sanidad Animal, Grupo de Investigación en Sanidad Animal y Zoonosis, Facultad de Veterinaria, Universidad de Córdoba, Córdoba, Spain; ^10^Department of Zoonoses, Faculty of Veterinary Medicine, Sohag University, Sohag, Egypt

**Keywords:** juice, probiotic, diabetes, functional properties, sensory attributes

## Abstract

The consumption of fruit and vegetable juices is widely recognized as a healthy choice across all age groups. Orange, carrot, and aloe vera are renowned for their functional properties and health benefits. In this study, we investigated the potential incorporation of aloe vera gel into blended orange and carrot juices. We also evaluated the resulting mixed probiotic juices (chemical, microbiological, and sensory aspects) during a 14-day storage period at refrigerator temperature. The chemical composition and phytochemical structure of aloe vera gel were examined, followed by an assessment of the biological effects of these healthy juices on diabetic albino rats. The results indicated improvements in total soluble solids, reducing sugars, and total sugars with increasing storage duration. Furthermore, the study demonstrated that incorporating aloe vera into the natural mixed juices enhanced their phytochemical quality. The treatment supplemented with aloe vera gel gave the highest total content of phenolic and flavonoid substances, which were 310 mg of GAE/100 g and 175 mg of quercetin/100 g, respectively. Probiotic strains (*Bifidobacterium animalis* subsp *lactis* Bb12, *Lactiplantibacillus plantarum* 299V, and *Lactobacillus acidophilus* L10) exhibited good viable cell counts in orange and mixed orange and carrot probiotics juices with viable counts of 7.42–8.07 log CFU/mL. Regarding sensory attributes, the study found that increasing the ratio of orange juice improved the taste while increasing the ratio of carrot juice enhanced the color in juice mixtures. Incorporation of aloe vera into mixed natural juices also enhanced the reduction of blood glucose, triglyceride, cholesterol, LDL, creatinine, ALT, AST, and urea levels while increasing total protein and HDL levels in diabetic rats. Based on these findings, oranges, carrots, and aloe vera offer the potential to produce new, flavorful, nutritious, and appealing juices. Moreover, this study determined that a functional juice with favorable sensory properties can be created by blending 75% orange juice, 20% carrot juice, and 5% aloe vera gel. Additionally, aloe vera demonstrated greater efficacy as an antidiabetic agent in rats. Further research is suggested to explore the potential advantages of aloe vera gel and probiotic juices in mitigating diabetes and other metabolic syndromes.

## 1 Introduction

Food components and functional foods play a crucial role in improving various health conditions beyond their nutritional value (1–12). This dietary choice encompasses various nutritional supplements, whole grains, vegetables, fruits, and beverages, all selected by individuals to enhance their health. Among others, beverages that reduce blood glucose by mixing fruit juices with low-sugar elements (vegetables) can be very beneficial through special biological and physical properties (13). Consuming vegetables and fruits is a substantial portion of a healthy diet, amongst other various food elements, and they are a superb provenance of bioactive ingredients (14). Orange and carrot juice can supply vitamins, polyphenols, and carotenoids, making them effective in preventing diabetes, heart disease, and cancer. Orange juice is made by squeezing fresh oranges and then undergoing a dehydration process. Oranges (*Citrus sinensis*) are rich in zinc, pectin, iron, manganese, chlorine, potassium, phosphorus, folic acid, and sodium (15). They have an enormous content of dietary fiber and are considered an excellent origin of bioactive ingredients such as carotenoids and phenolics (16–18). Oranges also have antioxidants, vitamins, and minerals. Vitamin C is necessary to protect the body from free radicals within cells (16, 19). More importantly, oranges are available in quantities throughout the year at an affordable price (20), and a glass of orange juice contains 112 g calories (21, 22). Carrots (*Daucus carota*) are rich in antioxidants (phenolic, flavonoids) and β-carotene (23, 24). They contain vitamin A, which is necessary for eye health and vision and reduces the risk of macular degeneration and cataracts that cause vision loss (25). Carrot juice is a ratable origin of carotenoids, vitamins, and minerals (21). Juice mixture is deemed a method that alleviates the nutritional quality of the juice product and also sensory characteristics (21). Prior research (26) has found that blending carrot juice with various fruits and vegetables improves overall nutritional characteristics and acceptability. Blending two or more fruits to create beverages offers a promising approach for developing new products with beneficial effects on sensory attributes, nutrition, and health (23).

Aloe vera (*Aloe barbadensis Miller*) is a perennial succulent shrub belonging to the family of *Asphodelaceae* (*Liliaceae*) (27–29). It is grown and cultivated in dry regions across the globe. Multiple studies have indicated that aloe vera can be an alternative to certain medications because it contains bioactive ingredients such as anthraquinones, anthrones, alkaloids, and others. These components contribute to its potent antidiabetic and anti-inflammatory properties (30, 31). Furthermore, aloe vera is an effective agent in combating most diseases (32) and can decrease blood glucose in diabetic patients (33). There are many vitamins, minerals, and antioxidants in aloe vera gel (34), which is used to enhance the nutritional quality of some juices and can be consumed because of its beneficial influence on health (35). Developing blended juices presents a viable option for enhancing the taste of aloe vera gel. Blending aloe vera gel with orange juice aims to enhance its physicochemical, microbial, and organoleptic properties (36). A previous study (37) evidenced that aloe vera gel has medicinal effects and contains bioactive polysaccharides, including mannose and glucose, known as “glucomannans.” Additionally, aloe vera juice contains significant amounts of xylose, arabinose, galactose, rhamnose, and numerous vitamins such as C, E, A, B1, B2, and niacin. However, aloe vera juice has low levels of magnesium, calcium, selenium, sodium, zinc, potassium, and manganese. In previous literature (38), fruit juices supplemented with probiotics have been considered functional drinks fortified with calcium and vitamins. Another study (39) revealed that fruit beverages might be a suitable medium for adding probiotics. Probiotics are defined as microorganisms that have a beneficial effect on health when taken in convenient doses (40).

The incorporation of functional foods to promote microbial balance and gut health has emerged as an innovative approach to reducing the risk of chronic illnesses. Extensive evidence suggests that supplementing food with probiotics can enhance health by modulating gut bacteria. Although numerous bacterial species are recognized as probiotics, *Lactobacillus* and *Bifidobacterium* spp. are the most extensively studied and have been incorporated into various food matrices to create a diverse range of functional products (41, 42). In this regard, *Lactiplantibacillus plantarum* (*L. plantarum*) is a microorganism commonly used in food fermentation technology and is generally considered safe. It also has applications in producing probiotic foods, including the commercially available *L. plantarum* 299v strain. This facultative heterofermentative lactic acid bacterium (LAB) exhibits remarkable resilience to conditions typically fatal to LAB, such as high acidity and ethanol concentrations. Notably, *L. plantarum* possesses unique characteristics, including its adaptability to various fermentation processes and metabolic versatility. Its ability to thrive in diverse environments is likely due to its relatively large genome size, averaging 3.3 Mb, which is one of the largest among the *Lactobacillus* genera. It should be noted that *L. plantarum* has been isolated from numerous food sources, including cereals, meats, dairy products, vegetables, fruits, beverages, and human and mammalian niches (43, 44).

Probiotics, also known as “good” bacteria, include *Bifidobacterium animalis* subsp. *lactis* (*B. lactis*), which produces lactic and acetic acids. These beneficial bacteria, such as *B. lactis*, play a role in food digestion and nutrient absorption, and defense against harmful organisms that can cause illness. *B. lactis*, a subspecies of *B. animalis*, is commonly found in probiotic supplements and the human gut. It is utilized to address various health issues such as respiratory tract infections, constipation, irritable bowel syndrome (IBS), and colic in newborns. Additionally, it is used to manage conditions such as diarrhea, hay fever, dental cavities, and many others, although scientific evidence supporting its efficacy in some cases is limited. For instance, it is worth noting that there is insufficient data to support the use of *B. lactis* for treating COVID-19.

Sometimes, *Bifidobacterium animalis* subsp. *lactis* is labeled as *Bifidobacterium lactis* or *B. lactis* on product packaging. It is crucial to differentiate *B. lactis* from other probiotics and fermented food products, such as yogurt, kefir, and fermented milk, as they are not interchangeable (45, 46). First identified in 1900, *Lactobacillus acidophilus* (*L. acidophilus*) is a rod-shaped, homofermentative, Gram-positive anaerobic bacterium commonly found in the human body, notably in the mouth, vagina, and digestive system, as well as in various fermented foods such as yogurt and milk. *L. acidophilus* thrives best in acidic environments with pH levels below 5.0, and its optimal growth temperature is 37°C. Certain commercial strains of *L. acidophilus* are utilized in dairy production due to their potent probiotic properties.

The genome sequencing of *L. acidophilus* has been completed, and it is known to inhibit the growth of harmful bacteria through antagonistic mechanisms (47, 48). Numerous previous studies have indicated that probiotics serve as a dietary supplement to support intestinal health and prevent gastrointestinal infections (49–51). Several previous works have investigated the impact of incorporating fermented plants or juices fermented by probiotic bacteria. In this context, a previous study (52) found that adding date juice to bio-yogurt made with a probiotic starter (*Bifidobacterium longum, L. acidophilus*, and *Streptococcus thermophilus*) affected its microbiological and physicochemical properties, with the viability of probiotic bacteria declining over a 21-day period; however, the bio-yogurt containing 10% date juice retained a higher count of probiotic bacteria compared to other samples. Another study (53) assessed the bacterial viability of different types of fermented onions utilizing probiotic starters (*Lactobacillus acidophilus* (LA-5), *Bifidobacterium bifidum* (BB-12), and *Streptococcus thermophilus* after a 24-h fermentation at 37°C and subsequent 28-day refrigeration. The findings revealed microorganism viability levels at Log 7.79 and 7.57 CFU/g.

Further, the literature also suggests that providing probiotic-fortified juice to individuals with lactose intolerance who cannot consume dairy products would be highly beneficial and practical. However, there is currently limited information available regarding the effects of fortifying healthy juices with aloe vera gel and probiotics. This includes understanding their impact on various physicochemical and organoleptic properties and their potential antidiabetic effects on albino rats. Therefore, this study aimed to blend aloe vera gel with orange and carrot juices to enhance their functional, physicochemical, and organoleptic properties while also investigating its potential antidiabetic effects on albino rats.

## 2 Materials and methods

### 2.1 Plant sources and phytochemicals composition of aloe vera gel

Fresh aloe vera leaves (*Aloe barbadensis Miller*) between 50 and 70 cm in length were acquired from the desert of Matrouh governorate, Egypt. The area is located 240 km (150 mi) west of Alexandria and 222 km from Sallum, along the main highway that connects the Nile Delta to the Libyan border. Oranges (*Citrus sinensis*), especially an assortment of baladi oranges at a full maturity phase, and carrots (*Daucus carrota* L) were brought from the local supermarket on the tenth day of Ramadan, in Egypt. The chemical and phytochemical composition of aloe vera gel was determined as reported elsewhere (54).

### 2.2 Microorganisms and their maintenance

The Phytone-Yeast Trypticase (TPY) medium contains (per liter(10 g of trypticase (BBL), 5 g of phytone (BBL), 5 g of glucose, 2.5 g of yeast extract (Difco), 1 ml of Tween 80, 0.5 g of L-cysteine HCl, 2 g of K_2_HPO_4_, 0.5 g of MgCl_2_ 6H_2_O, 0.25 g of ZnSO_4_ 7H_2_O, and 0.15 g of CaCl_2_. The TPY medium was combined with agar-agar at a concentration of 15 g/l to create TPY agar. The medium had a pH level of about 6.0. In 1 L of Beeren's agardium, there were 44 g of Columbia agar (Oxoid CM331), 5 g of glucose, 0.5 g of L-cysteine HCl, 5 g of agar-agar, and 5 mL of propionic acid. Propionic acid was added following sterilization of the medium, and 1N NaOH was used to bring the pH to 5.0 De Man–Rogosa–Sharpe (MRS) agar, which was prepared based on a recipe described elsewhere (55). The probiotic strains (*Bifidobacterium animalis subsp lactis* Bb12, *Lactiplantibacillus plantarum* 299V, and *Lactobacillus acidophilus* L10) were provided by Chr. Hansen A/S (Hrrsholm, Denmark).

### 2.3 Determination of probiotic viability during storage

The vitality of the probiotic *L. plantarum* in various juice samples was assessed using spread plating and serial dilution methods. Viable cell counts were performed during 2 weeks of chilled storage on days 1, 7, and 14. Juice samples were serially diluted up to 10^−7^ using 0.1% (w/v) sterile peptone water, and 100 μL of an appropriate dilution was spread in triplicate on MRS agar plates. Viable counts were determined after 48 h of anaerobic incubation at 37°C. In contrast, *B. lactis* and *L. acidophilus* required anaerobic conditions for growth, and this environment was created using the Bugbox anaerobic chamber (Ruskin Technology, USA). *B. lactis* was cultured on TPY agar, while *L. acidophilus* was cultured on MRS-sorbitol. Both were incubated at 37°C for 48–96 h (56). The colony-forming units were counted during fermentation using the plate counting method (57). Results for probiotic counts in juice samples were expressed as log CFU per mL.

### 2.4 Preparation of natural juices and phytochemicals composition of aloe vera gel

Oranges and carrots were chosen and washed carefully in tap water. Then, the fruits were cut into slices, and the juice was extracted by hand reamer (vegetable juicer). The extracted juice was then filtered directly and used for blending. All juices were pasteurized at 85°C for 10 min, and subsequently, pasteurized juices were warm-filled in sterilized and cleaned glass bottles (125 ml). The fruit juice was stored in anaerobic conditions at 37°C using an Anaerobe Jar + GasPak System (OXOID) or a Bugbox anaerobic chamber after being injected with Bifidobacterium or *Lactobacillus* bacteria. All samples were incubated with a 24-h-old probiotic culture (>105 CFU/mL) at 30°C for 72 h. *Bifidobacteria* and *Lactobacilli* colony-forming unit (cfu) counts were performed on samples at regular intervals. Additionally, the pH was determined. The juice had 1% of the fructo-oligosaccharide Raftiline added as a prebiotic during 0, 16, and 24 h of fermentation and preserved for 21 d at refrigerator temperature. Aloe vera gel was obtained by cutting leaves perpendicularly and mingled in a juice mixer to make it homogenized and smooth. Then, it was filtered through muslin cloth and stored at refrigerator temperature until use.

### 2.5 Preparation of juice treatments and its blends

In this study, six treatments were made of orange juice, carrot juice, and their mixtures, and the treatments were as follows:

- T1: 100% nature orange juice
- T2: 75% orange juice with 25% carrot juice
- T3: 95% orange juice with 5% aloe vera gel
- T4: 75% orange juice, 20% carrot juice and 5% aloe vera gel
- T5: 100% orange juice and probiotics
- T6: 75% orange juice, 25% carrot juice and probiotics.

All juice treatments were packaged in 200 ml sterile glass bottles and stored at refrigerator temperature until use.

### 2.6 Method of analysis

All juice blends were estimated chemically and microbiologically at zero time, then after 7 d of storage at refrigerator temperature. Sensory evaluation was carried out only at zero time. Changes in total and reducing sugars were evaluated (58). A total of 278 μL of diluted samples were put into 2 mL Eppendorf tubes. Following an extraction solvent, 278 μL of 5% aqueous phenol solution was added to the tubes. Thousand microliter of sulfuric acid was carefully added to each tube after a brief vortex agitation, adhering to all chemical safety requirements and utilizing the appropriate protective practices. Then, using a Power Wave XS 201595 spectrophotometer (BioTek Inc., Winooski, VT, USA) outfitted with a platereader (Biotek KcJunior), absorbance was recorded at 480 nm after 30 min. The calibration curve was made using galacturonic acid (GalA) (G212598%; Sigma-Aldrich, St. Louis, MO, USA). The data were given in units of g kg1DM. The 3,5-dinitrosalicylic acid technique was used to determine the amount of reducing sugar. GalA (0–5 mg mL−1) in methanol was used to create the calibration curve. At 540, absorbance was measured. The value of non-reducing sugars was calculated by deducting the reducing sugars from the total sugars. The changes in the brix value of all total soluble solids (TSS) in the juices were recorded by a digital refractometer (DR 6000, A. Kruss Optronic GmbH, Hamburg, and Germany). The changes in the pH value were measured by the glass electrode of a digital pH meter (Model Mettler Toledo, Switzerland), as described elsewhere (58). The color was measured by using the Hunterlab Colorflex (HunterLab, Reston, Va., US), as reported by Stinco et al. (59).

Total polyphenol content (TPC) was specified using the Folin-Ciocalteau system at 765 nm (UV-Vis spectrophotometer, Jenway, Staffordshire, UK), as illustrated by Gao et al. (60). The total phenol content was used as gallic acid equivalent in mg/L. In a test tube, 1.5 mL of Folin-Ciocalteu reagent and 0.2 mL of the juice were combined. 1.5 mL of a 6% sodium carbonate solution was added to the mixture after it had been incubated at room temperature for 5 min. The mixture was again incubated at room temperature for 90 min. A quartz cuvette was used to measure the blue color's absorption at 725 nm. The following gallic acid standards were created: 0.75 mg of gallic acid was dissolved in 5 mL of distilled water. Different amounts (0, 0.1, 0.2, and 0.3) of the gallic acid standard were pipetted into four marked test tubes. To finish the quantities to 0.3 mL, water was added. Later, 2.25 mL of Folin-Ciocalteu reagent was added, and the mixture was allowed to sit for 5 min at room temperature. The mixture was then re-incubated for 90 min at room temperature with 2.25 mL of a 6% sodium carbonate solution. The total phenolic content of the samples was calculated and expressed as milligrams of gallic acid equivalents (GAE) per 100 mL of sample. A standard curve was plotted using the absorbance of the resulting blue color, which was read using a quartz cuvette at 725 nm. The flavonoid content was determined spectrophotometrically, as mentioned elsewhere (61), which was then read at 510 nm. The value of total flavonoid was accurately determined in triplicate and expressed as quercetin equivalent in mg/L. AlCl3 test was used to assess the flavonoid content. In this step, a total of 300 μL of 5% NaNO_2_ was added to an aliquot of 500 μL of sample. Six hundred microliter of 10% after 5 min. After another 5 min, AlCl3 was added, and then 2 mL of 1 M NaOH was added to the mixture. Using a UV-Vis Agilent 8453 spectrophotometer (Agilent Technologies, Italy), the absorbance was measured at = 510 nm. The data were given as mg/100 mL of quercetin equivalents.

The sensory evaluation of natural mixed juices was assessed by 13 members of the staff of the Agri-Industrialization Unit, Desert Research Center (Cairo), and 15 members of the staff of the Food Science Department, Faculty of Agriculture, Zagazig University. A team of panelists was asked to estimate color, taste, odor, textures, and overall acceptability using a 10-point scale as described (62). The panelists were instructed to wash their mouths with low-sodium spring water during the sensory evaluation session, and they were encouraged to write down any criticisms of the tested products. Plain and treated juices were presented in plastic cups coded with three-digit random codes. Each cup contained 100 mL of juice samples freshly removed from the refrigerator. The sensory evaluation was conducted using a comparative test with fresh juices as a reference sample. The data were collected in specially designed ballots.

### 2.7 Biological experimental design

This study was conducted with the approval of the Institutional Animal Care and Research Unit, Zagazig University, Egypt (Approval no. ZU-IACUC/2/F/339/2022). A total of 30 male albino rats, each weighing 130–135 g, were purchased from the Agricultural Research Center of Giza, Giza, Egypt, and housed in wire cages in a 25°C environment. The rats were housed in solo stainless-steel cages in healthy laboratory conditions in the biology laboratory of the Faculty of Agriculture, Zagazig University. Rats were fed on a basal diet for 7 days (adaptation interval). The specially prepared (63) basal diet contained 12.5% casein, 10% corn oil, 4% salt mixture, 5% fiber, 1% vitamin mixture, 0.3% DL-methionine, 0.2% choline chloride, and completed to 100% by adding corn starch. Animals also had access to water throughout the duration of the experiment. The animals were divided into five groups. After the adaptation duration (7 days), the first group continued feeding on the basal diet and was considered a negative control (G1). The other four groups were diabetic rats and fed on a basal diet. The rats in the second group (G2) were fed a basal diet and considered a positive control. All rats except the control (negative and positive) received juices percent 100 mg/Kg body weight (64). The three other groups were allowed to be fed on juices as follows: G3: diabetic rats fed on 75% fresh orange juice and 25% fresh carrot juice; G4: diabetic rats fed on 75% orange juice, 20% carrot juice, and 5% aloe vera gel; and G5: diabetic rats fed on probiotic juice (75% orange juice, 25% carrot juice). The original treatments were a 75% orange juice mixture + a 25% carrot juice mixture, as it was the best treatment in terms of sensory acceptance by the arbitrators. The other two treatments were chosen to study the effect of supplementing the aforementioned original treatments with 5% aloe vera, compared to supplementation with probiotic bacteria with a known therapeutic effect. All groups were left throughout the duration of the experiment (45 days), and the amount of diet consummation was registered every day to compute the food intake, while body weight was recorded every week. The food efficiency ratio (FER) was calculated using the equation FER = body weight gain/food consumed “45 days” (65).

### 2.8 Induction of diabetes

To induce diabetes, male albino rats received a single intraperitoneal injection of alloxan monohydrate at a dose of 150 mg/kg body weight, following the described protocol (66). Rats were allowed 5% glucose solution to overcome severe hypoglycemia (67). Fasting blood glucose levels in rats were measured after 2 days. Rats were inspected for diabetes through glycosuria with a blood glucose level of 200–310 mg/dL 3 d following experimental induction. Blood samples were collected from the tip of the tail after 3 and 6 weeks of feeding and analyzed using the enzymatic kit method (68).

### 2.9 Biochemical analysis of blood samples

At the conclusion of the study period (45 days), rats underwent overnight fasting and were euthanized under complete anesthesia through intraperitoneal injection of ketamine (90 mg/kg) and xylazine (5 mg/kg). Following the careful separation of the abdominal skin from the thoracic cavity, blood was drawn from the posterior vena cava and placed into a serum separator tube. The sera were obtained by centrifuging the collected blood at 3,000 rpm for 10 min. Subsequently, the serum samples were stored at −20°C until further analysis. Estimation of Triglycerides, LDL, and total cholesterol was performed (68–71). Glucose was measured (72), ALT and AST were determined as mentioned by Reitman and Frankel (73), and the total protein was determined using the methods described earlier (74). Uric acid and creatinine were measured using the methods mentioned elsewhere (75)^1^.

### 2.10 Statistical examination

A statistical analysis was chosen to determine the impact of the treatment. All evaluations were approved in triplicate, and the data was described as mean. Significant differences (*p* < 0.05) were designed using Duncan multiple range tests (76). The mean values and their standard errors (SEM) are presented, and GraphPad Prism V5.0 software (GraphPad, San Diego, CA, USA) was employed for data visualization. A significance threshold of *p* < 0.05 was used for all statistical analyses.

## 3 Results and discussion

### 3.1 Chemical and phytochemicals composition of aloe vera gel

According to the present study, the amounts of moisture, ash, fat, protein, and crude fiber were 96.6, 0.26, 0.05, 0.16, and 0.20 (g/100 g), shown in Table 1. These values are within the scope of the research described elsewhere (54). Aloe vera gel contains 12.40 mg of GAE/100 mL of phenol and 98.80 mg of quercetin/100 mL of flavonoid. A previous study (77) estimated the phenol and flavonoid concentration to be 9.71 and 100.87 mg/lit extract.

**Table 1:** Chemical and phytochemical composition of aloe vera gel.

**Chemical constituents (g/100 g)**	
Moisture	96.6 ± 2.4
Fat	0.05 ± 0.01
Protein	0.16 ± 0.03
Crude fiber	0.20 ± 0.02
Ash	0.26 ± 0.05
**Phytochemicals**	
Total phenol content (mg of GAE/100 g)	12.40 ± 0.74
Total flavonoid content (mg of quercetin/100 g)	98.80 ± 1.7

### 3.2 Effect of aloe vera and probiotic on reducing, non-reducing, and total sugar contents of juices during storage interval

Table 2 depicts the effect of fortification of aloe vera and probiotic juices during the storage interval on the total sugar contents of the final product. It was noticed that the contents of non-reducing sugars decreased in all treatments after 7 days of storage, and then it increased again after 15 days of storage. On the contrary, the reducing sugar content increased for all treatments after 7 days of storage, and then it decreased again after 15 days of storage. The total sugar content increased during the storage periods in all treatments. It was also noted that the content of reducing, non-reducing, and total sugars decreased in the treatments containing probiotics compared to the rest of the treatments during all storage periods because of the activity of probiotic bacteria and their metabolites. Different lactic acid bacteria strains displayed their distinctive sugar metabolizing features and common traits for using sugars. Similar findings were noticed by Kelebek and Selli (78), who revealed that LAB increased the amount of glucose by producing a significant amount of lactic acid in the juice, creating a low pH environment, and accelerating the rate at which sucrose is hydrolyzed into glucose and fructose. Meanwhile, a previous study (79) observed that cell strain, fermentation substrate, and other parameters were strongly correlated with the consumption of sugar by microorganisms. These outcomes provided additional confirmation of the soluble sugars' metabolic properties during the digestion of the LAB. It appears that the diminution in non-reducing sugar is accompanied by an increase in reducing sugar in juices (80, 81). Results in Table 2 revealed an obvious increase in the reducing sugars of all the mixed juices due to the long storage interval, and this rise reached its extreme values at the end of the storage period at room temperature for the storage interval (81). It has been suggested that this increase was attributed to the change of sucrose in reducing sugars (glucose and fructose), which might be caused by the acidity of the juice; the longer storage interval, and the high temperature during the storage interval. In our study, there was an increase in the orange ratio, which led to an increase in reducing sugar content for all studied treatments, consistent with some previous works (82). Concerning the total sugars, the previously presented data illustrated approximately the same observation of reducing sugars. However, increasing the storage phase led to a marked rise in the total sugar content of all studied juice blends, where this increment reached the maximum values at the end of the storage period. The marked increase in total sugars might be attributed to the hydrolysis of polysaccharides such as starch, cellulose, and pectin.

**Table 2:** Effect of aloe vera and probiotics on the total sugar content of juices during storage interval.

**Treatments**		**Non-reducing sugars**			**Reducing sugars**			**Total sugars**		
		**Storage interval**			**Storage interval**			**Storage interval**		
		**First day**	**7 d**	**14 d**	**First day**	**7 d**	**14 d**	**First day**	**7 d**	**14 d**
Control juices	T1	2.15 ± 0.01^c^	1.33 ± 0.01^e^	2.14 ± 0.01^b^	5.7 ± 0.01^c^	6.72 ± 0.01^b^	6.32 ± 0.01^d^	7.85 ± 0.01^b^	8.04 ± 0.0^1^	8.44 ± 0.01^a^
	T2	2.04 ± 0.01^d^	2.3 ± 0.01^a^	2.23 ± 0.01^a^	5.3 ± 0.06^d^	5.43 ± 0.01^f^	6.12 ± 0.01^e^	7.24 ± 0.01^f^	7.72 ± 0.0^1^	8.34 ± 0.01^c^
Aloe vera juices	T3	2.03 ± 0.01^d^	1.83 ± 0.01^b^	2.06 ± 0.01^c^	5.81 ± 0.01^b^	6.22 ± 0.01^e^	6.57 ± 0.01^c^	7.84 ± 0.01^c^	8.05 ± 0.0^1^	8.64 ± 0.01^a^
	T4	1.67 ± 0.01^e^	1.56 ± 0.01^g^	1.49 ± 0.01^e^	6.33 ± 0.01^a^	6.95 ± 0.01^a^	7.22 ± 0.01^a^	7.99 ± 0.01^a^	8.51 ± 0.0^1^	8.71 ± 0.06^d^
Probiotic juices	T5	2.33 ± 0.01^a^	1.9 ± 0.06^c^	1.78 ± 0.01^d^	4.11 ± 0.01^f^	4.44 ± 0.01^g^	4.8 ± 0.06^g^	6.43 ± 0.01^i^	6.34 ± 0.0^1^	6.68 ± 0.01^e^
	T6	2.23 ± 0.01^b^	2.08 ± 0.01^h^	1.92 ± 0.01^h^	4.24 ± 0.01^e^	4.44 ± 0.01^g^	4.94 ± 0.01^f^	6.47 ± 0.01^g^	6.52 ± 0.0^1^	6.86 ± 0.01^f^
Sig.		^**^	^**^	^**^	^**^	^**^	^**^	^**^	^**^	^**^

^a–*c*^Means in the same column with the same classification with different superscripts differ significantly (*p* < 0.05); and ^**^p < 0.01. T1, 100% nature orange juice; T2, 75% orange juice, 25%carrot juice; T3, 95% orange juice and 5% aloe vera gel; T4, 75% orange juice, 20% carrot juice, and 5% aloe vera gel; T5, 100% orange juice and probiotics T6, 75% orange juice, 25% carrot juice fermented with probiotics.

### 3.3 Effect of aloe vera and probiotic on the total soluble solids contents of juices during storage period

Table 3 shows the total soluble solids (TSS) in aloe vera and probiotic juices during the storage interval. The data demonstrated a considerable rise in the TSS percentage for the control juice without additions in all storage intervals as compared with the other sample juice. The TSS decreased in juice-probiotic samples after 14 days, especially 75% orange juice, 25% carrot juice, and probiotics, due to sugar consumption. These present findings concur with several previous works (83, 84) that interpreted an increase of total soluble solids (TSS) due to the elevated amount of organic acid and polysaccharides subsidized with orange juice. Similarly, previous studies (36, 85) observed that the elevated total soluble solid values have a major role in preserving the taste and flavor of juice mixtures throughout the storage interval. Furthermore, another study (86) demonstrated that values might have been lower if TSS values were not modified to 12°Brix.

**Table 3:** Effect of aloe vera and probiotics on total soluble solids content in juices during the storage interval.

**Total soluble solids (°Brix)**				
**Treatments**		**Storage interval**		
		**First day**	**7 days**	**14 days**
Control juices	T1	14.2 ± 0.06^a^	14.3 ± 0.06^ab^	14.6 ± 0.06^b^
	T2	13.5 ± 006^c^	13.7 ± 0.06^c^	14.1 ± 0.06^c^
Aloe vera juices	T3	14.1 ± 0.06^a^	14.4 ± 0.06^a^	14.8 ± 0.06^a^
	T5	13.8 ± 0.06^b^	14.2 ± 0.06^b^	14.5 ± 0.06^b^
Probiotic juices	T5	13.4 ± 0.06^c^	13.8 ± 0.06^c^	13.34 ± 0.01^d^
	T6	12.2 ± 0.06^d^	12.5 ± 0.06^d^	12.85 ± 0.01^e^
Sig.		^**^	^**^	^**^

^a–*c*^Means in the same column with the same classification with different superscripts differ significantly (*p* < 0.05); and ^**^p < 0.01.

### 3.4 Effect of aloe vera and probiotics on the pH values of juices

**Figure 1:**
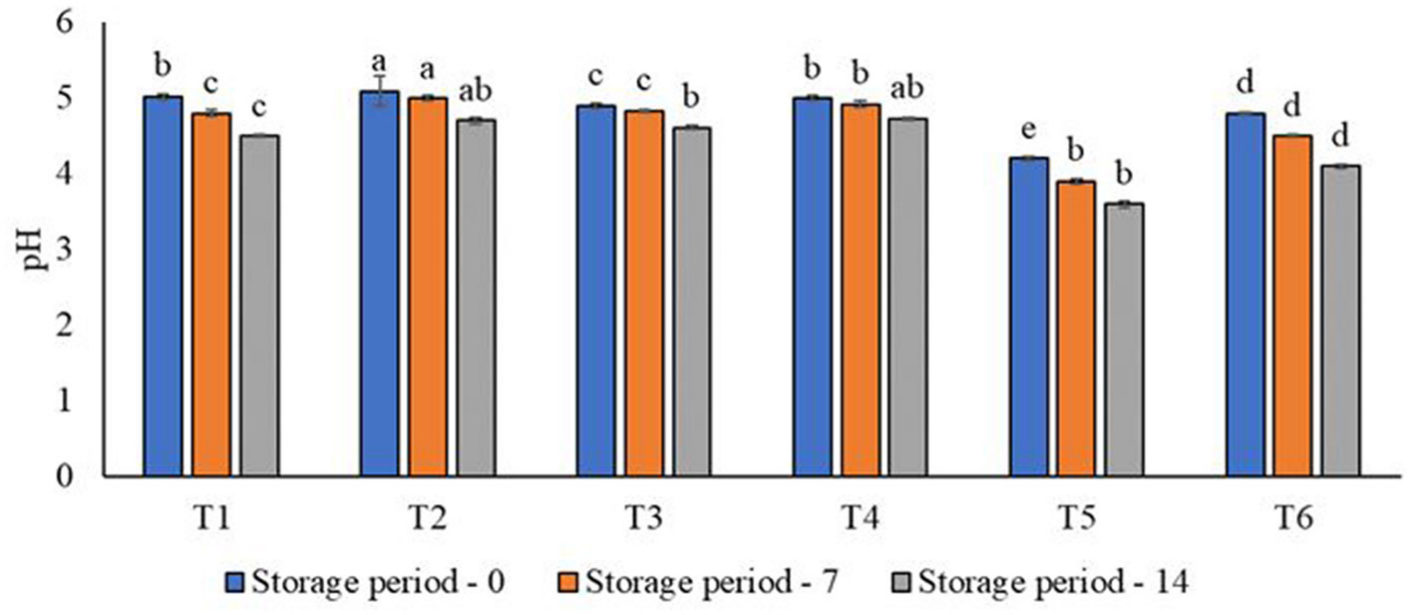
Effect of aloe vera and probiotic juice on the pH values during storage. T1: 100% natural orange juice; T2: 75% orange juice, 25% carrot juice; T3: 95% orange juice and 5% aloe vera gel; T4: 75% orange juice, 20% carrot juice, and 5% aloe vera gel; T5: 100% orange juice and probiotics; T6: 75% orange juice, 25% carrot juice fermented with probiotics. Each bar carrying different letters (a, b, c, d) is significantly different (*p* < 0.05).

The effect of aloe vera and probiotic juices on pH values during storage is shown in Figure 1. A decrease was observed in the pH values over the storage period. By the end of the storage period, the pH value of the control samples was 5.4 and 5.7. At the same time, it was 4.61 and 4.73 for the aloe vera gel-supported treatments, while the probiotic-supported treatments were 3.6 and 4.1, respectively. In the probiotic juice, there was a slight increase in the acidity values, corresponding to a slight decrease in the pH values. The present results are inconsistent with a previous study (87) that recorded no modulation in pH values of the various pasteurized juices of oranges and carrots at 4°C in the refrigerator and 10°C during the storage interval. One study (83) pointed out that three different formulations were created by blending carrot-orange juice, and the pH values decreased in all treatments during storage. Another study (36) pointed out that fresh orange-aloe vera juice treatments revealed no modulation in pH values during storage. A previous study (88) showed that probiotics reduced the pH of the juices during 10 h of incubation, either with or without encapsulated strains, due to the rise in acidity, which is consistent with our present findings (Figure 1). However, there were tenuous rises in pH values between the control and probiotic juices and a reduction during the storage from zero time (4.05) to 90 days (3.99). This reduction in pH may be due to the utilization of sugars present in juice by the probiotics to produce organic acids. Similarly, adding *Lactobacillus acidophilus* to carrot juice decreased the pH values of the resulting juice, as reported elsewhere (89).

### 3.5 Effect of aloe vera and probiotic on the color of juices during the storage interval

**Figure 2:**
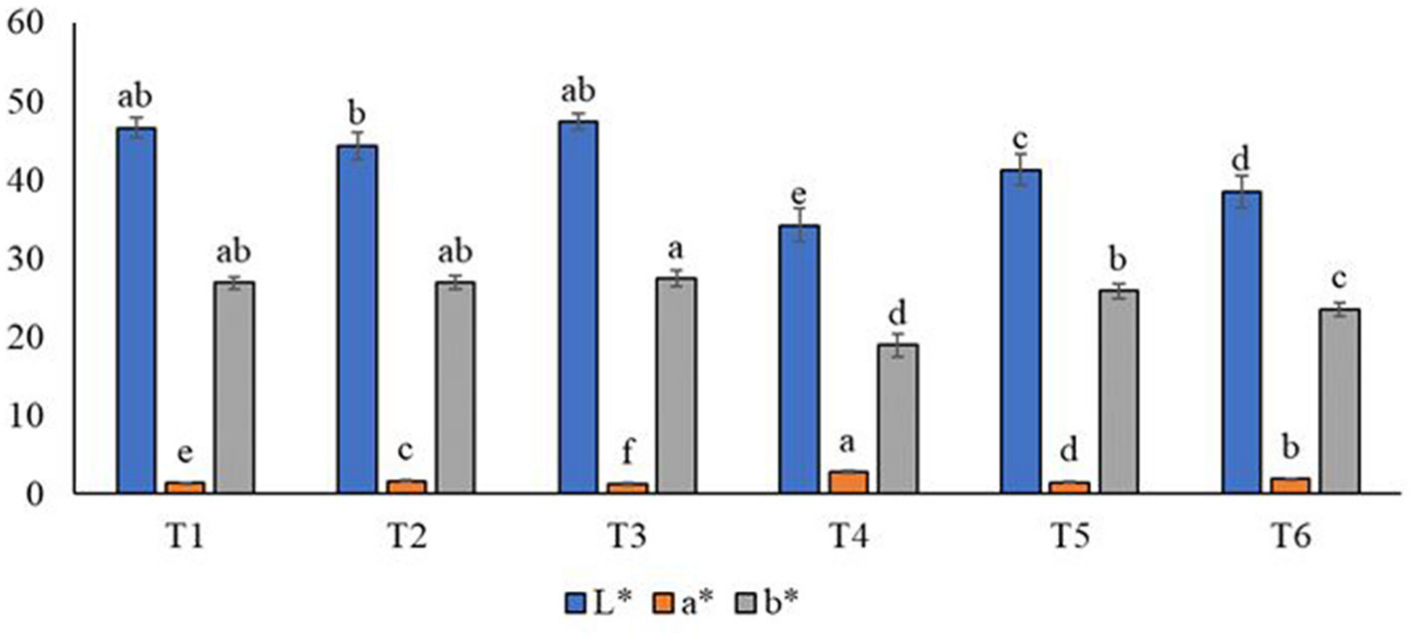
Effect of aloe vera and probiotics on color values of juice. T1: 100% nature orange juice; T2: 75% orange juice, 25% carrot juice; T3: 95% orange juice and 5% aloe vera gel; T4: 75% orange juice, 20% carrot juice and 5% aloe vera gel; T5: 100% orange juice and probiotics; T6: 75% orange juice, 25% carrot juice fermented with probiotics. Each bar carrying different letters (a, b, c, d, e, f) is significantly different (*p* < 0.05).

It should be stressed that color is one of the main indicators that show the changes in foods during the storage interval and significantly impacts consumer approval (90). As depicted in our study (Figure 2), control treatments experienced higher values of lightness L^*^ (47.39 and 46.67), as compared to T6 (38.49). In relation to variance, a^*^ and b^*^ values increased when the natural juices were subsidized with aloe vera gel. Another research project (91) illustrated that orange is excellent for the immune system, the heart, eyes, and cells. The data established that the aloe-gel addition ratio influences the taste and color. A previous study (92) indicated that the color estimate value decreases when the storage interval is increased. Another study (93) illustrated the color difference and concluded that the color was preferable at room temperature in the control treatment. However, the treated groups scored the least value at refrigerator temperature. Another study (94) indicated that the color variation in aloe vera gel at more than 4°C revealed a brown color at 25°C, including caramel anthraquinone oxidation, Maillard reaction, and ascorbic acid hydrolysis. On the other hand, color contrast in orange-carrot subsidized with aloe vera was decreased more than in the control sample at the end of the storage interval.

### 3.6 Effect of aloe vera and probiotic on the antioxidant content of juice during storage interval

The results of total phenol and flavonoid contents of aloe vera and probiotic juice during storage are illustrated in Table 4. It could be noted that control juices (without adding) on the first day and after 14 days had the highest level of total flavonoids and phenolics compared to the probiotic juices. The fortification of control juices with aloe vera gel increased the content of total phenols and total flavonoids compared to control juices. The rise in the antioxidant activity might have resulted from the hypothesis that polyphenols undergo polymerization reactions (95). A previous research study (96) revealed the rise in antioxidant activity, which is commonly attributed to Maillard interactions. In our study, the flavonoids in treatment 4 (mixed natural juice with aloe vera) were higher than in treatment 6 (mixed natural juice with probiotics), which was 180 mg/100 mL and 117.08 mg/100 mL, respectively. Polymerization reactions and the formation of new compounds led to unstable phenomena in flavonoids and phenols, including an increase and decrease during the storage interval. Our results are consistent with a previous study (97) that showed that most polyphenol compounds are found in aloe vera gel. The present findings are consistent with the study, which revealed that the flavonoid compounds of berry juices decreased during storage (98). Our study is in line with previous work (94), which documented an increase in the percentage of total phenol contents (TPC) and total flavonoid contents (TFC) of aloe vera juice at 25°C during storage for 30 days compared to storing it at 4°C. Other previous studies (36, 99) have also documented a prominent reduction in reducing the antioxidants of various fruits and vegetables at refrigeration temperature during storage intervals.

**Table 4:** Effect of total polyphenols (TPC) and flavonoids (TFC) content on aloe vera and probiotic juices during storage interval.

**Treatments**		**TPC mg/100 mL**			**TFC mg/100 mL**		
		**Storage interval (days)**			**Storage interval (days)**		
		**First day**	**7 day**	**14 days**	**First day**	**7 day**	**14 days**
Control juices	T1	330 ± 0.0^a^	340 ± 0.0^a^	300 ± 0.0^b^	200 ± 0.0^a^	190 ± 0.0^a^	175 ± 0.0^a^
	T2	280 ± 0.0^e^	305 ± 0.0^d^	320 ± 0.0^a^	140 ± 0.0^d^	130 ± 0.0^d^	115 ± 0.0^f^
Aloe vera juices	T3	310 ± 0.0^b^	310 ± 0.0^c^	270 ± 0.0^d^	190 ± 0.0^b^	175 ± 0.0^b^	170 ± 0.0^b^
	T4	300 ± 0.0^c^	320 ± 0.0^b^	250 ± 0.0^e^	180 ± 0.0^c^	165 ± 0.0^c^	160 ± 0.0^c^
Probiotic juices	T5	226.02 ± 0.01^f^	219.5 ± 0.06^f^	222.7 ± 0.06^f^	112.12 ± 0.01^h^	108.67 ± 0.01^h^	110.3 ± 0.06^g^
	T6	212.1 ± 0.06^g^	209.87 ± 0.01^g^	210.8 ± 0.06^g^	117.08 ± 0.01^g^	113.97 ± 0.01^g^	115.53 ± 0.01^e^
Sig.		^**^	^**^	^**^	^**^	^**^	^**^

^a–*c*^Means in the same column with the same classification with different superscripts differ significantly (*p* < 0.05); and ^**^p < 0.01.

### 3.7 Viable counts in the fermented orange juices and their mixtures

Cell viability is considered an important factor for the evaluation of functional products. Briefly, the three tested LAB strains revealed ~7.0 log CFU/mL under the same suitable conditions before addition into juices. All three LAB strains exhibited good viable cell counts in orange juices at pH 6.6, 37°C for 48 h, with viable counts of 7.42–8.07 log CFU/mL (Table 5). The result showed that orange juice, as a lactic fermentation substrate, could be beneficial for the growth of LAB, whose concentration was always higher than the minimum to maintain a healthy life (7.0 log CFU/mL) (100). Our results agree with data reported by Quan et al. (101), who found that orange juice could be beneficial for the growth of LAB. Also, the fortification of orange juice with carrot juice enhanced the viability of LAB strains compared with orange juice, possibly due to the lower acidity of carrot juice. Carrot juice also contains prebiotics that might improve the viability of LAB bacteria (102). The viability of LAB strains decreased as the storage period progressed in two treatments.

**Table 5:** Viable counts in the fermented orange juices during storage.

		**Probiotic juices**	
**Strains (log CFU/mL)**	**Storage period (day)**	**T5**	**T6**
*L. acidophilus*	1	7.54 ± 0.02^a^	7.86 ± 0.08^a^
	7	7.20 ± 0.11^b^	7.48 ± 0.06^b^
	14	6.88 ± 0.04^c^	7.02 ± 0.02^c^
*L. plantarum*	1	7.66 ± 0.24^a^	7.94 ± 0.14^a^
	7	7.30 ± 0.18^b^	7.58 ± 0.30^b^
	14	7.12 ± 0.12^c^	7.26 ± 0.16^c^
*B. lactis*	1	7.96 ± 0.18^a^	8.02 ± 0.30^a^
	7	7.72 ± 0.20^b^	7.88 ± 0.14^b^
	14	7.58 ± 0.26^c^	7.70 ± 0.24^c^

^a−−c^Means in the same column with the same classification with different superscripts differ significantly (*p* < 0.05).

### 3.8 Effect of aloe vera and probiotic on the sensory evaluation of juice during storage

The sensory properties of aloe vera and probiotic juice during the storage interval are illustrated in Table 6. The presented results showed that all samples were changed significantly in color, taste, odor, texture, and overall acceptability. The data in Table 6 indicates that the highest color score (9.5) was recorded for T2 and T4, followed by T6, which recorded a color score of (9.4). The results of the color parameter indicated that the carrot percent in natural mixed juices led to improved juice color compared with orange. From the same table, it could be observed that carrot percent led to a noticeable negative effect on the taste of the obtained natural mixed juices. Where the highest test scores (9.4) were recorded for T4, T5, and T6. Concerning flavor results, which are shown in Table 6, T5 had the highest flavor score (9.5), followed by T1 and T6 (9.4), while T2, T3, and T5 had the lowest flavor score (9.3). Regarding texture results presented in Table 6, all treatments had a slight effect on juice texture, where the scored values ranged between 9.3 and 9.5. In relation to overall acceptability results, T3, T4, and T6 have the highest overall acceptability (9.5), followed by T2 and T5 (9.4), while the lowest values were recorded for T1 and T3 (9.3). As depicted in Table 6, orange had a noticeable positive effect on the taste and flavor of the obtained natural mix. On the contrary, carrots had a noticeable positive effect on the color and textures of the obtained natural mixed. A previous study (36) demonstrated that adding aloe vera gel at the rate of 5% might be helpful in ameliorating taste, flavor, and overall acceptance of samples. During storage, there was a good sensory evaluation for aloe vera juice-orange juice for 45 days, which declined when the storage period reached 90 days. Another work (103) proved that the convenient mixing of vegetable and fruit juices modifies acidity and saccharides, besides promoting microbial inhibition, taste, bioactive component preservation, and odor of the final juice product. Another previous study (86), considering the overall acceptability of samples fortified with probiotics, concluded that there were no significant variances in sensual characteristics of freshly prepared probiotics juice samples after 10 h of incubation, while orange juice without probiotics cleared the lowest acceptance.

**Table 6:** Effect of aloe vera and probiotics on the sensory evaluation of juice.

**Treatments**		**Color**	**Taste**	**Odor**	**Textures**	**Overall acceptability**
Control juices	T1	9.3 ± 0.06^bc^	9.3 ± 0.06^a^	9.4 ± 0.06^ab^	9.3 ± 0.06	9.4 ± 0.06^a^
	T2	9.5 ± 0.06^a^	9 ± 0.06^b^	9.3 ± 0.06^bc^	9.4 ± 0.06	9.1 ± 0.06^b^
Aloe vera juices	T3	9.2 ± 0.06^c^	9 ± 0.06^b^	9.3 ± 0.06^bc^	9.3 ± 0.06	9.5 ± 0.06^a^
	T4	9.5 ± 0.06^a^	9.4 ± 0.06^a^	9.3 ± 0.06^bc^	9.5 ± 0.06	9.5 ± 0.06^a^
Probiotic juices	T5	9.3 ± 0.06^bc^	9.4 ± 0.06^a^	9.5 ± 0.06^a^	9.4 ± 0.06	9.4 ± 0.06^a^
	T6	9.4 ± 0.06^ab^	9.4 ± 0.06^a^	9.4 ± 0.06^ab^	9.5 ± 0.06	9.5 ± 0.06^a^
Sig.		^**^	^**^	^**^	NS	^**^

^a–*c*^Means in the same column with different superscripts differ significantly (*p* < 0.05); NS, not significant; and ^**^p < 0.01.

### 3.9 Impact of feeding aloe vera and probiotic juice on blood glucose level of diabetic rats

**Figure 3:**
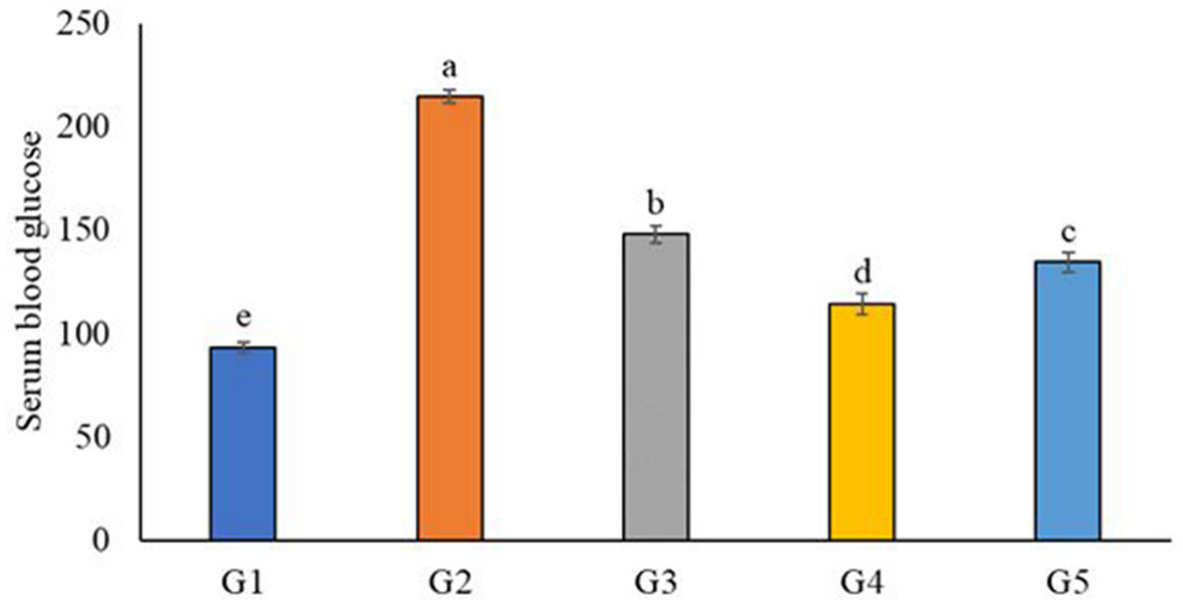
Impact of feeding aloe vera and probiotic juice on the serum blood glucose levels of diabetic rats. G1: negative control group fed on a basal diet; G2: positive control diabetic rats; G3: diabetic rats fed on 75% fresh orange juice and 25% fresh carrot juice; G4: diabetic rats fed on 75% orange juice, 20% carrot juice, and 5% aloe vera gel; G5: diabetic rats fed on 75% fresh orange juice and 25% fresh carrot juice fermented with probiotics. Each bar carrying different letters (a, b, c, d, e) is significantly different (*p* < 0.05).

Figure 3 shows the blood glucose level of diabetic rats that fed on functional natural juice subsidized with aloe vera and probiotics vs. the negative (–ve) and positive (+ve) control groups. In our study, the lowest glucose level reduction was in G4, which represented diabetic rats fed on 75% orange juice, 20% carrot juice, and 5% aloe vera (G4), and the blood glucose level was 114.27 g/dl. This was followed by G5, which was fed 75% orange juice, 25% carrot juice, and probiotics, and it had a blood glucose level of 134.63 g/dl. Similarly, a previous study (104) reported that the aloe vera gel reduced the blood glucose level of diabetic animals. These results concur with previous studies (33, 105–107), which confirmed that aloe vera significantly helps lower glucose sugar. A previous work (108) illustrated that probiotics were an active factor in reducing blood glucose levels and triglyceride in rats compared to the control group.

### 3.10 Body weights of diabetic albino rats feeding on aloe vera and probiotic juices

Table 7 depicts the feeding effect of diabetic rats on functional natural juice supplemented with aloe vera and probiotics on body weight gain (BWG), food intake (FI), and feed efficiency ratio (FER). As shown, the positive control group (–ve) had a higher rate of BWG (81 ± 0.58a), food intake (31 ± 0.58a), and feed efficiency ratio (0.06 ± 0.01a) when compared to the other groups. Meanwhile, diabetic rats fed functional natural juice supplemented with aloe vera and probiotics showed a significant decrease in these parameters compared to the positive control group, but this decrease was gradual. Furthermore, diabetic rats fed on functional natural juice supplemented with aloe vera and probiotics displayed significantly reduced body weight gain, food intake, and feed efficiency ratio compared to rats fed on a basal diet. A previous study (104) showed the oral administration of aloe vera juice decreased rats' body weight at the end of the experiment. It was found that the group orally given aloe vera juice gained the highest value of BWG, followed by the negative (healthy) control group, while the positive control group with diabetes decreased in weight due to type 2 diabetes. These findings are consistent with the main characteristics of diabetes, which include thirst, frequent eating, urinating, hyperglycemia, weight loss, and low insulin levels (109). The increase in the weight of animals treated with aloe vera juice could be attributed to the presence of magnesium, as it plays a major function in stabilizing lipid membranes, reproduction, and metabolic processes (110, 111).

**Table 7:** Body weight of diabetic rats feeding on aloe vera and probiotic juices.

**Groups**	**Body weight gain (g 60 day)**	**Food intake (g day)**	**Feed efficiency ratio**
G1 (–ve)	81 ± 0.58^a^	31 ± 0.58^a^	0.06 ± 0.01^a^
G2 (+ve)	71 ± 0.58^b^	28 ± 0.58^b^	0.06 ± 0.01^b^
G3	48 ± 0.58^c^	29.1 ± 0.06^b^	0.04 ± 0.01^c^
G4	27 ± 0.58^e^	29.3 ± 0.06^b^	0.03 ± 0.01^e^
G5	38 ± 0.58^d^	28 ± 0.58^b^	0.03 ± 0.01^d^
Sig.	^**^	^**^	^**^

^a–*c*^Means in the same column with the same classification with different superscripts differ significantly (*p* < 0.05); and ^**^p < 0.01. –ve, negative control group fed on a basal diet; +ve, positive control diabetic rats; G3, diabetic rats fed on 75% fresh orange juice and 25% fresh carrot juice; G4, diabetic rats fed on 75% orange juice, 20% carrot juice, and 5% aloe vera gel; G5, diabetic rats fed on 75% fresh orange juice and 25% fresh carrot juice fermented with probiotics.

### 3.11 Effect of aloe vera and probiotic juices on serum lipids profile diabetic rats

The results presented in Table 8 show that triglyceride and total cholesterol levels of the positive control group were significantly higher than the other experimental groups during the experimental intervals compared with the negative control group. In recent years, functional foods have become popular and are considered a curative and preventive agent for some chronic diseases such as diabetes. As shown in Table 8, feeding diabetic rats on aloe vera and probiotic juices caused a significant improvement in lipid profile. Our study illustrated that diabetic rats (G2 and G3) had the highest levels of TG, TC, LDL, and VLDL compared to the groups that feed on functional natural juices supplemented with aloe vera and probiotics (G4 and G5). Also, it could be observed that in groups G4 and G5, the value of HDL (35.40 and 32.60 mg/dl, respectively) was increased compared to groups G2 and G3. A previous study (34) noticed that all liver enzymes, blood cholesterol, HDL (high-density lipoprotein) cholesterol, and LDL (low-density lipoprotein) cholesterol reverted to almost normal levels because of the aloe vera gel. Feeding rats on aloe vera gel did not show any important alteration in total protein and albumin when compared with the control group.

**Table 8:** Effect of aloe vera and probiotics juices on serum lipids profile diabetic rats.

**Groups**	**Triglycerides (mg/dl)**	**Total cholesterol (mg/dl)**	**HDL (mg/dl)**	**LDL (mg/dl)**
G1	83.70 ± 3.40^d^	80.60 ± 3.35^d^	40.50 ± 1.70^a^	24.98 ± 1.64^e^
G2	132.30 ± 2.66^a^	100.20 ± 4.70^a^	28.60 ± 1.40^d^	45.14 ± 2.02^a^
G3	108.20 ± 2.80^b^	93.50 ± 2.90^b^	30.50 ± 1.701^b^	41.54 ± 2.60^b^
G4	95.80 ± 2.70^b^	87.90 ± 2.70^b^	35.40 ± 1.50^c^	33.34 ± 1.20^d^
G5	102.30 ± 2.30^c^	91.90 ± 2.50^c^	32.60 ± 1.60^c^	38.84 ± 1.94^c^
Sig.	^**^	^**^	^**^	^**^

^a–*c*^Means in the same column with the same classification with different superscripts differ significantly (*p* < 0.05); and ^**^p < 0.01. –ve, negative control group fed on a basal diet; +ve, positive control diabetic rats; G3, diabetic rats fed on 75% fresh orange juice and 25% fresh carrot juice; G4, diabetic rats fed on 75% orange juice, 20% carrot juice, and 5% aloe vera gel; G5, diabetic rats fed on 75% fresh orange juice and 25% fresh carrot juice fermented with probiotics.

### 3.12 The kidney and liver functions of diabetic albino rats

Table 9 shows the impact of aloe vera and probiotic juices on the kidney and liver functions of diabetic rats. Concerning kidney function, feeding diabetic rats on aloe vera and probiotic juices (G4 and G5) showed a significant reduction in serum creatinine and urea levels compared with G2 and G3. Furthermore, the positive control group (+ve) illustrated an important rise in ALT and AST enzyme compared with the other groups. Similarly, a previous study (104) illustrated the impact of oral management of aloe vera and probiotic juice on the significant increase in blood urea level of the animals of the group given aloe vera juice compared to the control groups. The same finding was noticed in the significant value of creatinine for the animals of the group given aloe vera juice. A previous report (104) pointed out the influence of oral administration of aloe vera juice on the significant values of liver enzymes for animals of the group given aloe vera juice, whereas GOT significantly decreased in the treated group (46.33 mg/dl) when compared to the infected group (58.33 mg/dl).

**Table 9:** Effect of aloe vera and probiotic juices on the Kidney and liver functions of diabetic rats.

**Groups**	**Kidney functions**		**Liver functions**		

	**Creatinine (mg/dl)**	**Urea (mg/dl)**	**Total protein (g/dl)**	**ALT (U/L)**	**AST (U/L)**
G1	0.52 ± 0.01^e^	19.53 ± 0.01^d^	5.44 ± 0.01^a^	37.25 ± 0.01^e^	27.15 ± 0.01^c^
G2	0.94 ± 0.01^a^	31.53 ± 0.01^a^	3.75 ± 0.01^e^	67.96 ± 0.01^a^	54.99 ± 0.01^a^
G3	0.80 ± 0.01^b^	29.3 ± 0.06^b^	3.84 ± 0.01^d^	53.22 ± 0.01^c^	45.44 ± 0.01^b^
G4	0.73 ± 0.01^c^	24.5 ± 0.01^c^	4.64 ± 0.01^c^	50.25 ± 0.01^b^	22.46 ± 0.01^d^
G5	0.58 ± 0.01^d^	14.5 ± 0.01^e^	5.37 ± 0.01^b^	41.46 ± 0.01^d^	22.5 ± 0.06^d^
Sig.	^**^	^**^	^**^	^**^	^**^

^a–*c*^Means in the same column with the same classification with different superscripts differ significantly (*p* < 0.05); and ^**^p < 0.01. –ve, negative control group, fed on a basal diet; +ve, positive control diabetic rats; G3, diabetic rats fed on 75% fresh orange juice and 25% fresh carrot juice; G4, diabetic rats fed on 75% orange juice, 20% carrot juice and 5% aloe vera gel; G5, diabetic rats fed on 75% fresh orange juice and 25% fresh carrot juice fermented with probiotics.

## 4 Conclusions

The study demonstrated the possibility of using aloe vera gel and probiotics in orange and carrot juices, in addition to evaluating the functional element of the juices (chemically, microbiologically, and sensory) during 14 days of storage at refrigerator temperature. The present study also revealed the improvement in the physicochemical, microbiological, and sensory properties of healthy juices containing aloe vera gel and probiotics. This was reflected by the improvement of total soluble solids, reducing sugars and total sugars, and the phytochemical quality with good and acceptable organoleptic properties. Moreover, the study confirmed that adding aloe vera to the natural mixed juices improved sensory attributes. The study revealed that increasing the orange juice ratio improved the taste, whereas increasing the carrot ratio improved the color. Given the abovementioned results, oranges, carrots, and aloe vera can be used to produce new, tasteful, and healthy juices. In addition, this research concluded that aloe vera juice is more efficacious as an antidiabetic agent in diabetic rats. Collectively, the study pointed out that using orange and carrot juice enriched with aloe vera might be promising for treating diabetes, while orange and carrot juice fortified with probiotics could also help overcome lactose intolerance. Further research on the mechanistic pathways underlying these antidiabetic effects of healthy juices containing aloe vera gel and probiotics should be studied.

## Data availability statement

The original contributions presented in the study are included in the article/supplementary material, further inquiries can be directed to the corresponding authors.

## Ethics statement

This study was conducted with the approval of the approval of the institutional animal care and research Unit, Zagazig University (Institutional Review Board Number ZU-IACUC/2/F/339/2022). The study was conducted in accordance with the local legislation and institutional requirements.

## Author contributions

SM: Conceptualization, Data curation, Formal analysis, Investigation, Methodology, Project administration, Validation, Visualization, Writing – original draft, Writing – review & editing. AA-N: Formal analysis, Investigation, Methodology, Software, Supervision, Validation, Writing – original draft. ER-Á: Funding acquisition, Project administration, Resources, Software, Visualization, Writing – review & editing. FA: Data curation, Investigation, Software, Supervision, Validation, Visualization, Writing – original draft. HS: Formal analysis, Investigation, Software, Supervision, Validation, Visualization, Writing – original draft. ME-K: Data curation, Funding acquisition, Resources, Software, Validation, Writing – review & editing. MS: Data curation, Funding acquisition, Resources, Software, Validation, Visualization, Writing – review & editing. AH: Funding acquisition, Resources, Software, Writing – review & editing. AAA: Data curation, Funding acquisition, Resources, Software, Validation, Visualization, Writing – review & editing. AA: Data curation, Funding acquisition, Resources, Software, Validation, Visualization, Writing – review & editing. EE: Data curation, Formal analysis, Funding acquisition, Investigation, Resources, Software, Supervision, Validation, Visualization, Writing – original draft, Writing – review & editing.

## Footnotes

^1^Standard A, Sample A. Creatinine. Jaffé. Colorimetric–Kinetic. Ref.: Jaf-005.
